# Who Gained Insurance Coverage in 2014, the First Year of Full ACA Implementation?

**DOI:** 10.1002/hec.3349

**Published:** 2016-04-08

**Authors:** Charles Courtemanche, James Marton, Aaron Yelowitz

**Affiliations:** ^1^ Department of Economics, Andrew Young School of Policy StudiesGeorgia State UniversityAtlantaGAUSA; ^2^ Department of Economics, Gatton College of BusinessUniversity of KentuckyLexingtonKYUSA

**Keywords:** Affordable Care Act, insurance coverage, Medicaid, private insurance, Marketplace coverage

## Abstract

The most significant pieces of the Affordable Care Act (exchanges, subsidies, Medicaid expansion, and individual mandate), implemented in 2014, were associated with sizable gains in coverage nationally that were divided equally between gains in Medicaid and private coverage. These national trends mask heterogeneity in gains by state Medicaid expansion status, age, income level, and source of coverage. © 2016 The Authors. *Health Economics* published by John Wiley & Sons Ltd.

## 1 Introduction

Significant pieces of the Affordable Care Act (ACA), including Medicaid expansions, federal subsidies for the purchase of policies in state and federal Marketplaces, and the individual mandate, were implemented in 2014. Because of lags in the dissemination of survey data, it is only just now possible to report changes in insurance coverage both nationally and for various sub‐groups between 2013 and 2014.

This paper uses the 2013 and 2014 waves of the American Community Survey (ACS) to characterize changes in insurance coverage nationally, as well as by state, age, and percent of the Federal Poverty Level (FPL). We consider a detailed set of sources of insurance coverage: Medicaid, Medicare, Veteran's Administration, Tricare, employer‐provided, and individual coverage. The ability to stratify individuals and sources of coverage is important because we expect the ACA to have varying impacts on coverage sources, depending on personal characteristics and state choices. For example, one would expect a larger increase in coverage for poor adults in states that expanded Medicaid as compared with non‐expansion states.

Nationally, the uninsured rate fell from 14.8 to 12.0%, a reduction that was divided equally between gains in Medicaid and private coverage. The largest gain in coverage occurred in Kentucky (5.7 percentage points), while the smallest occurred in Wyoming (0.3 percentage points). By age, the largest gains in coverage were for non‐elderly adults, especially those aged 19 to 25 (5.3 percentage points). By income, the largest gain (5.5 percentage points) occurred for those with family income between 100 and 137% of the FPL. The source of these gains varied.

## 2 Study Data and Methods

We used data from the ACS, conducted by the Census Bureau, to examine insurance changes between 2013 and 2014. The ACS is an excellent data source because of its large sample size, mandatory respondent participation (unlike the Current Population Survey (CPS) or the Behavioral Risk Factor Surveillance System (BRFSS)), breadth of questions on sources of insurance, focus on contemporaneous coverage, and uniformity of questions over time. 1
http://www.census.gov/programs‐surveys/acs/methodology/mandatory‐voluntary‐methods.html
 Unlike the BRFSS, the ACS has several distinct categories of health insurance sources. The ACS focuses explicitly on current coverage, leading to less confusion than the CPS questions, which ask about coverage in the previous calendar year. The CPS question is sometimes misinterpreted by respondents as asking about current coverage. 2Swartz K. Interpreting the estimates from four national surveys of the number of people without health insurance. Journal of Economic and Social Measurement. 1986; 14(3):233–242. Importantly, the CPS insurance questions were redesigned in 2014, increasing the difficultly in making over‐time comparisons. 3
https://www.census.gov/hhes/www/hlthins/data/incpovhlth/2013/redesign.html
 One drawback with the public version of the ACS is that the respondent's interview date within the year is unknown. However, virtually all changes from the ACA occurred on January 1, limiting the need for precise interview timing. 4Michigan's expansion started in April 2014. See: http://kff.org/health‐reform/state‐indicator/state‐decisions‐for‐creating‐health‐insurance‐exchanges‐and‐expanding‐medicaid/



We classify those who were elderly (or disabled) and report being on both Medicare and another plan as Medicare beneficiaries. We distribute the small remaining fraction with multiple sources of coverage evenly across reported sources of coverage, such that the totals add up to 100%. None of these assumptions affect our calculation of insurance gains.

One confounding factor is that the national economy was improving. The unemployment rate fell from 8.0 to 5.6% between January 2013 and December 2014, and it also fell in every state. 5
http://data.bls.gov/timeseries/LNS14000000
 An improving labor market would likely lead to gains in employer‐provided coverage.

## 3 Potential for Gains in Coverage because of the ACA

The ACS measures household income relative to the FPL and the respondent's age. As is illustrated in Table 1, the federal ACA provisions, along with state choices, imply different potential sources of coverage gains for individuals depending on their age and income. For the elderly, we expect very little change, because virtually all qualify for Medicare. Poor children were virtually all previously eligible for Medicaid. 6Yelowitz A. The Medicaid notch, labor supply and welfare participation: evidence from eligibility expansions. The Quarterly Journal of Economics. 1995; 110(4): 909–939. However, the individual mandate may have compelled parents to enroll their children who were previously “conditionally covered”. 7Marton J, Yelowitz A. Health insurance generosity and conditional coverage: evidence from Medicaid managed care in Kentucky. Southern Economic Journal. 2015; 82(2):535–555. For children above the FPL, the mandate and other insurance market changes would be expected to raise coverage.

**Table 1:** Sources for insurance coverage gains

		Individual mandate binding	New Medicaid eligibility because of ACA	Subsidized individual coverage premiums	Subsidized individual coverage cost sharing	Guaranteed issue and community rating	Existing public coverage (Medicaid, CHIP, or Medicare)
Age 0–18							
0–99% FPL, Expansion		✓					✓
0–99% FPL, Non‐expansion		✓					✓
100–137% FPL, Expansion		✓					✓
100–137% FPL, non‐expansion		✓					✓
138–249% FPL		✓		✓	✓	✓	
250–399% FPL		✓		✓		✓	
400% + FPL		✓				✓	
Age 19–64							
0–99% FPL, Expansion		✓	✓				
0–99% FPL, Non‐expansion						✓	
100–137% FPL, Expansion		✓	✓				
100–137% FPL, Non‐expansion		✓		✓	✓	✓	
138–249% FPL		✓		✓	✓	✓	
250–399% FPL		✓		✓		✓	
400% + FPL		✓				✓	
Age 65+							
0–400% + FPL		✓					✓

Notes: FPL stands for Federal Poverty Level, and Expansion and Non‐expansion refer to whether an individual's state expanded the Medicaid program under the ACA. The FPL was $11 670 for individuals in 2014 and was $23 850 for a four‐person household. Insurance gains for adults aged 19 to 64 assume that the individual did not previously qualify for Medicaid via Supplemental Security Income, pregnancy expansions, Temporary Assistance for Needy Families, the Medically Needy program, or state‐specific adult expansions.

For non‐elderly adults, coverage gains and sources are expected to vary depending on income. In non‐expansion states, poor adults were not entitled to a federal subsidy, but could purchase community‐rated individual Marketplace coverage. 8For expansion status, see: http://kff.org/health‐reform/state‐indicator/state‐activity‐around‐expanding‐medicaid‐under‐the‐affordable‐care‐act/
 In Medicaid expansion states, such individuals would be eligible for Medicaid. Thus, the majority of the expected increase in coverage for poor adults would likely come from Medicaid in expansion states and private coverage in non‐expansion states. Given differences in premiums, we expect the increase in coverage in expansion states to be larger for poor adults.

Adults with incomes falling between 100 and 137% of the FPL were eligible for Medicaid in expansion states and were eligible for large federal subsidies for Marketplace coverage in non‐expansion states. 9For an adult at the FPL, the expected contribution towards coverage for the second lowest silver plan would be 2 percent of income. The voucher amount could then be applied to any plan. Thus, one would expect sizable gains for both groups, but from different sources. For those with incomes falling between 138 and 399% of the FPL, Marketplace purchases were subsidized on a sliding scale, so gains should come from private coverage. Finally, for those with higher incomes, the individual mandate compels coverage, but purchases were not subsidized. An individual for whom insurance was deemed affordable could elect not to purchase coverage and instead pay a penalty, which was relatively low in 2014. 10
https://www.healthcare.gov/fees/fee‐for‐not‐being‐covered/



The subsequent tables characterize actual changes in coverage levels and sources between 2013 and 2014 and compare those changes to expectations described earlier.

## 4 Results

Table 2 lists the percentage uninsured nationally and by state, as well as the percentage covered by each source for 2013 and 2014. Nationally, the uninsured rate fell from 14.8 to 12.0%, a 2.8 percentage point reduction that was divided equally between gains in Medicaid and individual coverage. The largest gains occurred in two states that opted to expand Medicaid, Kentucky (5.7 percentage points) and Nevada (5.2 percentage points). These increases were largely driven by gains in Medicaid coverage. Kentucky experienced a 4.3 percentage point increase in Medicaid coverage, and Nevada experienced a 3.7 percentage point increase. The smallest coverage gains occurred in Wyoming and Massachusetts. Factors ranging from the choice not to expand Medicaid, strong local economies, and prior health insurance reforms likely explain the modest gains for states such as these. 11
http://kff.org/medicaid/report/trends‐in‐medicaid‐and‐chip‐eligibility‐over‐time/

12Courtemanche C, Zapata D. Does universal coverage improve health? The Massachusetts experience. Journal of Policy Analysis and Management. 2014; 33(1): 36–69.


**Table 2:** Changes in sources of insurance coverage, 2013 to 2014 full US population

	Uninsured		Medicaid		Medicare		VA		ESHI		Individual coverage		Tricare	
	2013	2014	2013	2014	2013	2014	2013	2014	2013	2014	2013	2014	2013	2014
US	14.8%	12.0‡	14.1	15.3‡	15.8	16.2‡	0.6	0.6†	46.8	47.1‡	6.3	7.2‡	1.6	1.6†
AL	14.1	12.4‡	14.3	14.9†	18.2	18.4	0.7	0.7	43.7	44.8‡	6.8	6.7	2.2	2.1
AK	18.3	17.5	11.5	12.5	9.6	10.1	1.3	1.4	47.3	44.9‡	4.6	5.4†	7.4	8.1
AZ	17.8	14.1‡	15.5	17.2‡	16.6	17.2‡	0.8	0.7	41.3	41.9†	6.5	7.1‡	1.5	1.7‡
AR	16.2	12.0‡	17.0	19.4‡	18.9	18.9	1.0	1.0	39.7	40.6†	5.6	6.8‡	1.7	1.3‡
CA	17.3	12.7‡	16.3	18.9‡	13.5	13.9‡	0.5	0.4†	44.1	44.8‡	7.2	8.1‡	1.2	1.2
CO	13.9	10.6‡	11.7	14.4‡	13.5	13.8	0.7	0.7	48.3	48.7	8.6	8.9	3.2	2.9‡
CT	9.3	6.9‡	13.9	15.9‡	16.5	16.6	0.4	0.4	52.8	52.4	6.0	6.8‡	1.1	0.9†
DE	10.5	7.9‡	15.9	15.3	18.2	17.9	0.4	0.6†	49.4	51.6‡	4.2	4.8	1.5	2.0‡
DC	6.3	5.7	20.9	22.7†	12.4	11.8	0.6	0.4	50.6	49.3	7.8	8.6	1.3	1.3
FL	20.4	17.0‡	13.2	13.7‡	20.1	20.6‡	0.7	0.8‡	36.9	37.2	6.7	8.9‡	2.0	1.9
GA	19.3	16.4‡	13.4	14.2‡	13.7	14.4‡	0.8	0.7	44.3	45.0‡	5.7	6.7‡	2.7	2.6
HI	7.1	4.9‡	12.2	13.2†	16.4	16.6	0.6	0.7	49.0	50.5†	5.7	5.3	9.1	8.8
ID	16.5	13.8‡	11.7	11.5	15.5	15.9	1.1	0.8‡	45.2	45.1	8.5	11.2‡	1.6	1.7
IL	12.9	10.0‡	15.0	16.1‡	14.8	15.1†	0.4	0.5	50.3	50.7	5.9	7.0‡	0.6	0.7
IN	14.4	12.4‡	12.5	12.9†	16.1	16.5†	0.6	0.6	50.2	51.0‡	5.6	6.0‡	0.6	0.6
IA	8.9	5.9‡	12.6	13.6‡	17.4	17.5	0.6	0.5	52.7	52.8	7.2	9.0‡	0.6	0.7
KS	12.6	10.9‡	10.1	10.9‡	15.8	16.2	0.7	0.6	50.5	50.4	7.3	8.3‡	3.0	2.7†
KY	14.9	9.2‡	14.1	18.5‡	17.6	18.3‡	0.8	0.7†	45.8	45.6	5.1	6.1‡	1.7	1.7
LA	17.5	15.3‡	16.7	16.9	15.5	16.1†	0.6	0.6	42.2	42.7	5.9	6.6‡	1.5	1.8‡
ME	11.1	9.8‡	16.3	15.6	20.3	20.9	0.9	0.7†	44.6	45.9†	5.1	6.0‡	1.6	1.2‡
MD	10.3	8.2‡	12.3	14.1‡	14.3	14.8†	0.6	0.6	53.9	53.1†	6.2	6.9‡	2.5	2.3
MA	3.9	3.5‡	16.6	17.8‡	16.0	16.3	0.3	0.3	56.1	55.5†	6.5	6.1‡	0.5	0.5
MI	11.3	8.7‡	15.4	15.9‡	17.3	18.1‡	0.6	0.5	49.1	49.8‡	5.8	6.4‡	0.6	0.6
MN	8.4	6.1‡	11.7	13.1‡	15.1	15.6†	0.6	0.6	55.7	55.7	8.0	8.3	0.5	0.6
MS	17.6	15.2‡	18.1	18.4	17.3	17.6	0.8	0.7	38.7	39.5†	5.4	6.6‡	2.2	1.9†
MO	13.3	11.8‡	11.7	11.2‡	17.5	18.0†	0.8	0.7	48.3	48.8	6.6	7.8‡	1.8	1.6
MT	16.8	13.8‡	11.9	12.1	17.8	18.7	1.2	1.0	42.0	42.4	8.2	10.4‡	2.1	1.7
NE	10.7	9.4‡	9.8	9.9	15.5	15.7	0.6	0.6	52.0	52.2	9.2	10.0‡	2.1	2.2
NV	20.8	15.6‡	9.4	13.1‡	14.7	15.4†	1.0	0.8	47.5	47.0	4.9	6.3‡	1.8	1.8
NH	10.9	9.4‡	8.9	8.3†	16.9	17.5	0.6	0.6	56.3	56.7	5.6	6.5‡	0.8	1.1‡
NJ	13.5	11.2‡	11.0	12.3‡	15.6	15.7	0.2	0.2	54.0	54.3	5.1	5.7‡	0.6	0.4‡
NM	19.5	15.2‡	19.8	22.8‡	17.3	17.4	0.9	0.9	35.1	35.9	4.9	5.4†	2.5	2.3
NY	10.8	8.7‡	18.2	19.3‡	15.8	16.1†	0.3	0.3	48.5	48.5	5.7	6.6‡	0.5	0.5†
NC	15.5	13.1‡	14.5	14.6	16.6	17.0‡	0.7	0.7	42.2	42.8‡	7.2	8.4‡	3.3	3.3
ND	10.3	8.0‡	6.9	7.8†	14.9	15.3	0.6	0.4	54.1	55.3	10.1	11.0	3.2	2.1‡
OH	11.3	8.7‡	13.9	15.7‡	17.0	17.5‡	0.6	0.6	51.2	51.4	5.2	5.3	0.8	0.7†
OK	18.0	15.6‡	14.1	13.9	16.5	17.0	1.1	0.9†	42.2	43.1†	6.0	7.3‡	2.1	2.2
OR	15.0	10.1‡	13.4	18.3‡	17.2	17.7	0.9	0.8†	45.9	45.0†	6.9	7.2	0.6	0.8‡
PA	10.0	9.0‡	12.5	12.6	18.1	18.5†	0.5	0.5	52.1	51.3‡	6.2	7.6‡	0.6	0.5
RI	11.9	7.0‡	12.4	17.1‡	17.7	17.9	0.3	0.6‡	50.3	49.1	6.4	7.3†	1.1	1.0
SC	16.0	13.9‡	14.5	15.3‡	17.7	18.4‡	0.8	1.0†	42.9	42.2†	5.2	6.3‡	2.8	2.9
SD	12.6	10.4‡	11.3	10.5	16.5	16.8	1.0	1.1	46.0	47.8†	10.5	11.4	2.2	2.1
TN	14.1	12.2‡	15.2	15.7†	17.4	17.7	0.7	0.8†	44.2	44.1	6.3	7.5‡	2.1	2.0
TX	22.4	19.6‡	14.1	14.0	12.5	12.9‡	0.7	0.7	42.9	44.3‡	5.4	6.6‡	1.9	1.9
UT	13.6	12.6‡	8.9	8.6	10.8	11.1	0.5	0.3‡	56.8	57.0	8.0	8.9‡	1.4	1.4
VT	7.0	4.7‡	19.9	19.9	18.9	19.7	0.5	0.5	47.4	47.3	5.4	7.2‡	0.9	0.7
VA	12.4	10.9‡	8.7	9.0	15.0	15.2	0.7	0.8†	50.4	50.5	6.7	7.6‡	6.1	6.1
WA	14.1	9.3‡	12.1	14.8‡	15.1	15.6‡	0.8	0.7	48.3	48.8†	6.8	7.7‡	2.8	3.0†
WV	13.9	9.4‡	15.1	19.9‡	21.3	21.4	1.0	0.9	44.7	44.3	3.3	3.4	0.8	0.7
WI	9.3	7.7‡	13.3	13.2	16.7	17.0	0.6	0.6	53.5	53.8	5.9	7.0‡	0.6	0.6
WY	12.7	12.5	8.8	8.8	14.4	14.9	1.3	1.1	53.8	52.7	6.4	7.9‡	2.5	2.1

‡ Year‐by‐year difference is significant at 1% level.

† Year‐by‐year difference is significant at 5% level. See text for how we dealt with multiple sources of coverage.

Table 3 aggregates coverage into three categories (uninsured, public, and private coverage) and stratifies by age and income. Among age groups, the largest coverage gains occurred for those aged 19 to 25 (5.3 percentage points). These young adults had a 1.9 percentage point increase in public coverage and a 3.4 percentage point increase in private coverage. The large gains among young adults are interesting because they already received a sizeable insurance expansion in 2010 with the mandate for private insurers to allow dependents to remain on parents' coverage until age 26. 13Sommers B, Buchmueller T, Decker S, Carey C, Kronick R. The Affordable Care Act has led to significant gains in health insurance and access to care for young adults. Health Affairs. 2013; 32: 165–174. The largest gain by income category occurred for those with incomes between 100 and 137 of the FPL (5.5 percentage points). The gain in public (private) coverage for this group was 3.5 (1.9) percentage points. There was no change in coverage for the elderly.

**Table 3:** Changes in coverage, 2013 to 2014, by age group and income group

	Uninsured		Public		Private	
	2013	2014	2013	2014	2013	2014
By Age						
Age 0–18	7.5%	6.3%‡	35.6%	36.4%‡	56.9%	57.3%‡
Age 19–25	25.8%	20.5%‡	12.0%	13.9%‡	62.2%	65.6%‡
Age 26–64	19.9%	16.1%‡	13.6%	15.3%‡	66.6%	68.6%‡
Age 65+	1.0%	1.0%‡	96.3%	96.3%	2.7%	2.8%‡
By poverty level						
FPL 0–99%	25.8%	21.4%‡	55.5%	58.6%‡	18.8%	20.0%‡
FPL 100–137%	25.3%	19.8%‡	49.9%	53.4%‡	24.8%	26.7%‡
FPL 138–249%	20.8%	16.9%‡	35.0%	37.2%‡	44.2%	45.9%‡
FPL 250–399%	12.2%	9.8%‡	23.0%	24.4%‡	64.8%	65.8%‡
FPL 400%+	5.1%	4.1%‡	16.8%	17.6%‡	78.1%	78.4%‡

‡ Year‐by‐year difference is significant at 1% level.

† Year‐by‐year difference is significant at 5% level.

Table 1 suggests that we expect the biggest potential gains in coverage to come from near‐poor non‐elderly adults, although the source is expected to vary based on state Medicaid expansion status. Table 4 examines this by stratifying by expansion status. There were increases in coverage of 8.8, 10.5, and 7.5 percentage points for adults with income below the FPL, between 100 and 137% of the FPL, and between 138 and 249% of the FPL, respectively, in expansion states. In non‐expansion states, the increases in coverage within these same groups are 4.7, 7.0, and 4.7 percentage points. Thus, the coverage gains were larger in expansion states within each of these income categories. 14Coverage gains for expansion states may have exceeded that of non‐expansion states at income levels above Medicaid eligibility because of differences in state attitudes about the ACA, among other reasons. As predicted, the majority of the increase in coverage for these adults in the expansion states came from public coverage, while the majority of the gain in coverage for these adults in non‐expansion states came from private coverage. This is true even for adults in the 138 to 249% of the FPL range, where we might have expected gains in private coverage to outweigh gains in public coverage in all states.

**Table 4:** Changes in coverage, 2013 to 2014, by Medicaid expansion status

	Uninsured		Public		Private	
	2013	2014	2013	2014	2013	2014
Expansion states						
*Children*						
FPL 0–99%	8.0%	6.3%‡	78.3%	79.8%‡	13.7%	13.9%
FPL 100–137%	10.2%	7.7%‡	65.2%	67.5%‡	24.7%	24.8%
FPL 138–249%	9.2%	6.9%‡	40.2%	43.0%‡	50.7%	50.1%†
FPL 250–399%	5.6%	4.3%‡	15.3%	17.1%‡	79.1%	78.6%†
FPL 400%+	2.3%	2.0%‡	4.7%	4.9%†	93.0%	93.1%
*Adults*						
FPL 0–99%	34.3%	25.5%‡	41.4%	49.0%‡	24.3%	25.5%‡
FPL 100–137%	37.1%	26.6%‡	32.5%	41.2%‡	30.4%	32.2%‡
FPL 138–249%	30.0%	22.5%‡	17.7%	22.5%‡	52.4%	55.0%‡
FPL 250–399%	16.8%	12.6%‡	8.6%	10.9%‡	74.6%	76.5%‡
FPL 400%+	6.3%	4.8%‡	3.5%	4.2%‡	90.2%	91.0%‡
Non‐expansion states						
*Children*						
FPL 0–99%	11.0%	9.7%‡	76.0%	77.5%‡	13.0%	12.8%
FPL 100–137%	12.8%	11.6%‡	62.2%	62.6%	24.9%	25.8%†
FPL 138–249%	11.9%	10.8%‡	36.2%	37.8%‡	51.9%	51.4%
FPL 250–399%	7.5%	6.7%‡	13.1%	13.7%‡	79.4%	79.5%
FPL 400%+	3.5%	2.9%‡	5.0%	5.0%	91.5%	92.1%‡
*Adults*						
FPL 0–99%	44.1%	39.4%‡	31.1%	32.6%‡	24.8%	27.9%‡
FPL 100–137%	43.3%	36.3%‡	24.0%	25.4%‡	32.7%	38.3%‡
FPL 138–249%	32.7%	28.0%‡	13.4%	14.0%‡	53.9%	58.0%‡
FPL 250–399%	17.8%	15.1%‡	7.0%	7.2%‡	75.2%	77.7%‡
FPL 400%+	7.3%	6.0%‡	3.5%	3.5%	89.2%	90.5%‡

‡ Year‐by‐year difference is significant at 1% level.

† Year‐by‐year difference is significant at 5% level.

For higher income adults, we still see gains in coverage in both expansion and non‐expansion states despite decreasing federal subsidies that phase‐out by 400% of the FPL. These adults may be responding to the mandate or benefiting from the other insurance market reforms. These increases were generally higher in expansion states.

We observe gains in coverage in both expansion and non‐expansion states for children, especially poor children. Among poor children who were already Medicaid‐eligible, coverage increased by 1.7 (1.3) percentage points in expansion (non‐expansion) states. These increases are driven by increased formal participation in Medicaid, as opposed to ‘conditional coverage’, in which parents might wait until their Medicaid‐eligible child needs care to formally enroll them. This change could potentially be attributed to the mandate. We also see gains in coverage for children in families whose income (100–249% of the FPL) would have likely already qualified them for CHIP coverage.

## 5 Discussion

A recent analysis of National Health Interview Survey focuses only on young adults and finds significant gains in coverage. 15McMorrow S, Kenney GM, Long SK, Anderson N. Uninsurance among young adults continues to decline, particularly in Medicaid expansion states. Health Affairs. 2015; 34 (4): 616–620. Our findings are consistent with this, although our purpose is to consider both broad national trends for all ages, as well as differences by state, age, income, and source of coverage. Overall, our results are generally consistent with our expectations regarding where the pieces of the ACA implemented in 2014 would have the largest impact.

The 2.8 percentage point national gain in coverage we observe was divided equally between gains in Medicaid and private coverage. We observe larger gains in Medicaid coverage for low‐income non‐elderly adults in states made newly eligible for Medicaid, while we see larger gains in private coverage for low‐income non‐elderly adults eligible for federal subsidies in non‐expansion states. We see gains in public coverage even among children whose family income already qualified them for public coverage.

Approximately 12% of the US population remained uninsured in 2014. One might expect additional gains in coverage as the economy continues to improve, as more states expand Medicaid, and as the size of the uninsured penalty increases. However, administration changes in several states, including most notably Kentucky, may cause states to revisit their expansion decisions or future plans. Thus, complete and timely data on changes in patterns of coverage will continue to be of interest to policymakers.

